# Development and validation of film stimuli to assess empathy in the work context

**DOI:** 10.3758/s13428-021-01594-6

**Published:** 2021-06-07

**Authors:** Cornelia Wieck, Susanne Scheibe, Ute Kunzmann

**Affiliations:** 1 Faculty of Behavioural and Social Sciences, University of Groningen, Grote Kruisstraat 2/1, Groningen, 9712 TS Netherlands; 2 Institute of Psychology, University of Leipzig, Neumarkt 9–19, 04109 Leipzig, Germany

**Keywords:** Empathy, Measure, Test development, Age, Work

## Abstract

A growing body of research suggests that empathy predicts important work outcomes, yet limitations in existing measures to assess empathy have been noted. Extending past work on the assessment of empathy, this study introduces a newly developed set of emotion-eliciting film clips that can be used to assess both cognitive (emotion perception) and affective (emotional congruence and sympathy) facets of empathy in vivo. Using the relived emotions paradigm, film protagonists were instructed to think aloud about an autobiographical, emotional event from working life and relive their emotions while being videotaped. Subsequently, protagonists were asked to provide self-reports of the intensity of their emotions during retelling their event. In a first study with 128 employees, who watched the film clips and rated their own as well as the protagonists’ emotions, we found that the film clips are effective in eliciting moderate levels of emotions as well as sympathy in the test taker and can be used to calculate reliable convergence scores of emotion perception and emotional congruence. Using a selected subset of six film clips, a second two-wave study with 99 employees revealed that all facet-specific measures of empathy had moderate-to-high internal consistencies and test–retest reliabilities, and correlated in expected ways with other self-report and test-based empathy tests, cognition, and demographic variables. With these films, we expand the choice of testing materials for empathy in organizational research to cover a larger array of research questions.

## Introduction

Due to technological advancements and increased globalization, the nature of work has changed (Greenhaus & Kossek, 2014). Employees, for example, experience enhanced interdependence with their team members and report needing a greater variety of skills to perform their work successfully (Wegman et al., 2016). Furthermore, the share of employment in service and health care sectors is continuing to rise (Eichhorst & Buhlmann, 2015). Owing to these changes, most contemporary professional roles require a high degree of empathy, that is, the understanding and sharing of others' emotions and experiencing sympathy with them. Consistently, the bulk of empirical studies suggests that empathy is a predictor of important work outcomes at individual and organizational levels (e.g., Cropanzano et al., 2016; Longmire & Harrison, 2018).

Despite increased interest in empathy, organizational researchers continue to debate the adequate measurement of empathy (Clark et al., 2019). Among the most frequent criticisms are that empathy is mostly measured through self-report questionnaires that often have been criticized for lacking validity, as they are vulnerable to impression formation biases and introspective limits (e.g., Dunning et al., 2004; Gerpott et al., 2020). Thus, scholars have repeatedly argued that self-report questionnaires should be complemented by more objective performance-based tests (e.g., Côté, 2014; Wieck & Kunzmann, 2015). In response to these calls, several performance-based tests have been developed (e.g., Nowicki & Duke, 1994; Schlegel et al., 2014; Young et al., 2002). Yet, a closer look at these tests reveals that many are far removed from real-life (job) situations in which empathy is enacted or empathic responses are elicited. Typically, these tests use highly artificial stimuli, such as static pictures of faces or short film clips, each presenting a particular emotion. Such stimuli are unlikely to evoke emotions in the test taker and do not present authentic emotions as they occur in meaningful contexts. Thus, there is a need to develop more ecologically valid measures of empathy to advance research in organizational settings.

The goals of the current paper were threefold. The first goal was to develop a new battery of film stimuli that specifically deal with real-life events in work contexts, evoke authentic emotions in the test takers, and allow assessing empathy in vivo. The second goal was to initially validate these newly produced film stimuli. To do so, we administered 26 film clips to a heterogeneous sample of employees and tested their (psychometric) properties including difficulty, internal consistency, and emotional potency. The third goal was to select a subset of six film clips with adequate psychometric properties and present them in a two-wave online study to an independent sample of employees to test the internal consistency, test-retest reliability, and construct validity of the film set.

### Defining and measuring empathy

Empathy is defined as a multidimensional construct composed of both affective and cognitive aspects (Cuff et al., 2016; Davis, 1996; Walter, 2012). The cognitive aspect – often labeled as emotion perception, emotion recognition, or empathic accuracy – describes the ability to correctly infer another person’s emotional state from verbal and nonverbal information such as facial expressions, vocal tones, or body postures (Elfenbein & Eisenkraft, 2010; Ickes, 1997). The affective aspect of empathy refers to emotional congruence or affective match, defined as the vicarious experience of another person’s emotional state (Beadle & de la Vega, 2019; de Vignemont & Singer, 2006). Emotional congruence is considered a double-edge sword. While constantly sharing the other persons’ suffering can trigger personal distress – a self-focused, aversive emotional reaction – or burnout (Batson et al., 1987; Petitta et al., 2017), a moderate level of attunement is necessary for effective functioning in professional life. For instance, emotional congruence allows interaction partners to share emotional experiences and experience collective affect, facilitating effective service encounters and organizational commitment, and thus has adaptive value (Andreychik, 2019; Gountas et al., 2014). Some researchers have adopted a broader definition of empathy that also includes sympathy, defined as an other-oriented emotion such as sorrow, concern, and warmth (Losoya & Eisenberg, 2001; Preckel et al., 2018). Although some scholars equate emotional congruence with sympathy, it is useful to distinguish the two as sympathy does not necessarily require sharing the interaction partner’s emotions (Batson et al., 1987). Consequently, it poses little risk for personal distress and burnout. In addition, sympathy, but not necessarily emotional congruence, triggers prosocial behavior (Eisenberg, 2003).

Extant organizational research either employed self-report scales or, to a much lesser extent, test-based measures to assess empathy. Whereas trait self-report measures typically assess both aspects of empathy (e.g., Interpersonal Reactivity Index, IRI, Davis, 1983), test-based measures have focused on emotion perception (e.g., Diagnostic Assessment of Non-verbal Abilities, DANVA, Nowicki & Duke, 1994; Geneva Emotion Recognition Test, GERT, Schlegel et al., 2014; Facial Expressions of Emotion Stimuli and Tests, FEEST, Young et al., 2002; see Dziobek et al., 2008 for an exception). An obvious reason for this focus is that the typical emotion perception tests present emotions in static, unimodal, and posed ways and thus do not evoke emotions in the respondent. As a result, emotional congruence and sympathy can hardly be assessed. A related problem is that most of these tests present emotions in a decontextualized manner, raising the question of whether the evidence from them can be generalized to real-life contexts (e.g., Blanke et al., 2015; Kunzmann & Isaacowitz, 2017).

Notably, to the best of our knowledge, there is only one experimental paradigm to date that allows both cognitive and affective facets of empathy to be assessed simultaneously and in vivo using dynamic and multimodal stimuli.^1^ In this paradigm, termed EmpaToM, Kanske and colleagues (2015) confront test takers with brief film clips (~ 15 s) of diverse actors reporting on events that vary in emotionality (either negative events or neutral events). Although this approach moves closer to an ecologically valid assessment of empathy, the fact that professional actors were utilized to narrate and emotionally portray events strictly specified by the authors, rather than experiencing and expressing genuine emotions, remains an important limitation. Moreover, this task only includes negative and neutral events, thus neglecting the diversity of emotional quality as it occurs in real-life empathic encounters.

### The new film stimuli to assess empathy in the work context

To complement and extend existing measures of empathy, we developed a set of stimuli that differ from available empathy tasks in three aspects: (1) the stimuli cover autobiographical work-related situations as recalled by employees, (2) the stimuli are emotionally potent eliciting a range of self- and other-related emotions in the test taker, and (3) the stimuli showcase a diversity of protagonists in terms of age and gender. With these three aspects, we take into account that most emotion-arousing situations at the workplace trigger not only one, but all facets of empathy that prototypically facilitate each other (e.g., sharing another’s emotions should help correctly perceiving this person’s emotions and vice versa). To depict this interplay among the dimensions of empathy and, at the same time, determine the relative predictive value of each dimension for work and organizational outcomes, the tasks need to be designed to elicit emotions and to embed emotions into meaningful contexts.

#### Tasks with work-related situational contexts

To develop tasks that are tailored to the work context, we invited a heterogeneous sample of employees into our laboratory and instructed them in individual sessions to relive and think-aloud about emotionally intense work events from their life while they are being video-taped. Compared to most extant empathy tasks, the resulting film clips convey dynamic and multimodal information from verbal (lexical expressions), visual (facial expressions), and auditory (prosodic expressions) channels, so that all of the test taker’s senses are addressed and the unfolding emotional process can be captured in vivo. In addition, the film clips cover work-related situations and, thus, allow investigating empathy contextualized to those settings for which organizational research wishs to predict outcomes. We consider this critical as past research has suggested that empathic responding is context-specific and dependent on the personal relevance as well as familiarity with the task (e.g., Katzorreck & Kunzmann, 2018; Zhang et al., 2013). Surprisingly, most of the available test-based measures in the organizational literature – particularly those of emotion perception – either fail to use work-related materials (e.g., DANVA) or only use it partially (e.g., Geneva Emotional Competence test, GECo, Schlegel & Mortillaro, 2019). However, the work context differs from non-work contexts in manifold ways, including a strong focus on performance within a definite period of time (often under time pressure), more explicit rules for employees' emotion expression and behavior towards interaction partners, and less control over interaction partners. Individuals who can correctly infer the emotional state of their in-laws, therefore, are not necessarily more empathic in interacting with colleagues and clients.

#### Tasks that are emotionally potent

A second goal of our approach has been to elicit a wide range of authentic emotional reactions in the test taker. Given that empathy is typically required and called upon in emotionally intense situations, in our view, a valid measure of empathy should be emotionally potent and successfully evoke emotions in the test taker. Yet, a closer look at prior literature revealed that the only available measure capable of eliciting emotional reactions in the test takers limits to empathic reactions towards negative episodes (Kanske et al., 2015). Although often playing a subordinate role, however, (working) life also encompasses sharing other people's positive emotions such as being vicariously proud of a successfully completed project by a coworker or being happy with a patient whose operation went well. In addition, research shows that positive and negative experiences are not symmetrical, and that the valence of a stimulus can have an impact on individuals' empathic responses and competencies. For example, it has been shown that people are more likely to share and perceive others' emotions when embedded in positive compared to negative narrations (e.g., Richter & Kunzmann, 2011). Consequently, it is necessary to provide a balanced number of positive and negative emotion-evoking stimuli in empathy tasks. We thus took care that protagonists talked about various work-related events ranging from very positive to very negative experiences.

#### Tasks with protagonists of diverse age and gender

The workforce is ageing steadily as a result of demographic and socio-economic changes (Toossi, 2012). Consequently, companies and their customers are becoming increasingly age-diverse, and workers find themselves collaborating and interacting with young, middle-aged, and older coworkers or clients. This raises two interrelated questions; first, whether there are differences in empathy of workers of different age (i.e., age of the perceiver), and second, whether it is easier for workers to infer others’ feelings if they are of the same or different age (i.e., age of the protagonist). While age-comparative research on empathy is comparatively new in I/O psychology, lifespan developmental research suggests that there are multidirectional age differences in empathy, with decreases in emotion perception and stabilities or even increases in emotional congruence and sympathy (e.g., Sze, Gyurak, et al., 2012b; Wieck & Kunzmann, 2015). Strikingly, the commonly used measures in organizational literature, especially those for emotion perception, have included stimuli depicting young (some also middle-aged) adults, but failed to include older adults. This possibly creates disadvantages for older test takers, leading to an underestimation of their empathic capacities. There is in fact evidence showing that older adults perform better when confronted with people of their own age describing problems that they are familiar with (Ebner et al., 2011; Katzorreck & Kunzmann, 2018; Wieck & Kunzmann, 2015). Moreover, perceiving emotions is generally more difficult in older compared to young faces, for instance, due to age-related changes in facial structure and physiognomic features (e.g., wrinkles, folds) or negative age stereotypes and implicit attitudes towards the elderly (e.g., Fölster et al., 2014).

In terms of gender, a substantial body of research has shown that women are more emotionally expressive than men and are therefore likely to be easier to read (e.g., Fischer & LaFrance, 2015), which may lead to higher levels of empathic responses and competencies towards them. Using diverse testing material is therefore crucial to more accurately capture empathy of workers of all ages and both genders.^2^

To sum up, with our film clips, we seek to extend organizational researchers’ toolkit with a measure of empathy that covers empathic responses and competencies as they occur in vivo and in response to authentic work-related situations.

## Study 1

The goals of Study 1 were twofold. The first goal was to produce a new set of film stimuli that specifically deal with emotionally charged real-life events in work contexts as recalled by diverse employees, evoke positive and negative emotional reactions in the test takers, and allow the simultaneous assessment of different facets of empathy in vivo. The second goal was to validate these newly produced stimuli by testing their (psychometric) properties including difficulty and internal consistency, and their emotional potency. Furthermore, we examined the effects of the protagonists’ characteristics and participants’ characteristics (i.e., age and gender, respectively), as well as film clips’ valence on the three facets of empathy.

In line with previous work employing the same film-based paradigm (e.g., Katzorreck & Kunzmann, 2018; Kunzmann et al., 2018; Wieck & Kunzmann, 2015), we expected the newly developed tasks to be moderately difficult and emotionally potent, allowing us to capture individual differences and to avoid ceiling effects in the three aspects of empathy. Assuming that emotion perception, emotional congruence, and sympathy ideally mutually influence each other and together facilitate adaptive functioning, we further predicted positive intercorrelations among the three empathy facets, particularly between the two emotional facets (Wieck & Kunzmann, 2015). Regarding protagonists’ demographic variables, in line with the few studies on empathy, we expected to find an advantage for the test takers’ empathy if the film clips depict women (compared to men) or young protagonists (compared to middle-aged and older protagonists; e.g., Fölster et al., 2014). Consistent with prior work (Beadle et al., 2015; Sze, Gyurak, et al., 2012b; Wieck & Kunzmann, 2015), we further predicted that participants' age is negatively related to emotion perception, while age is unrelated to emotional congruence and even positively related to sympathy. Finally, as to the film clips’ valence, we expected that emotional congruence and sympathy are more easily elicited if test takers are confronted with positive rather than negative narrations. This pattern was expected to hold for emotion perception, insofar as participants’ recognition ability would be better when protagonists report positive, as compared to negative, situations.

### Method

#### Development of film stimuli

##### Film production

A group of 46 employed adults from the community (hereafter called “protagonists”) ranging in age from 20 to 64 years (*M* = 38.91, *SD* = 11.58, 52.2% women) was recruited in a midsize German city via newspaper ads, flyers, and from the department’s participant pool. Protagonists had to be at least 18 years old and work a minimum of 20 h a week. They received monetary compensation of 8 euros/hour. All protagonists were Caucasian and German native speakers.

In order to develop a new set of emotion-eliciting stimuli, we employed a paradigm often used in emotion research, the relived emotion task (e.g., Kunzmann et al., 2017; Levenson et al., 1991; Wieck & Kunzmann, 2015). In this paradigm, protagonists were asked to recall and relive emotional memories from their working life. In order to adapt to the laboratory setting and the film recording, protagonists first described a neutral situation (the route they had taken to the lab). In subsequent semi-structured interviews, protagonists were guided by the interviewer to remember and to select a suitable work situation that they recently experienced to relive. The situation had to be (a) either emotionally positive or negative, (b) emotionally complex insofar as the protagonist experienced a mix of one primary emotion and one or two secondary emotions of the same valence (e.g., higher-intensity sadness in combination with lower-intensity anger, or higher-intensity happiness in combination with lower-intensity pride), and (c) recently experienced (i.e., during the last year).

Protagonists were instructed to focus on the most important aspect of the situation and told that their thinking-aloud should have a clear beginning and ending. In addition, the protagonists were asked to speak out loud everything that came to their mind and that they themselves find important and relevant about the situation, but to focus on their own thoughts and actions and to not refer to the specific feelings that they had experienced during the situation. This last instruction was required because we wanted to use the resulting film clips to assess emotion perception.

After protagonists had selected a suitable event, the interviewer left the room. Guided by computer-based instructions, protagonists were given as much time as needed to sort their thoughts and remember details of the event before they quietly relived the emotions for 10 s. Subsequently, they started to think-aloud about the emotion-evoking situation while being videotaped. To standardize the material, protagonists were instructed to avoid extreme movements or face-masking and to look at the screen while retelling the event. If the protagonist remembered more than one suitable work event (preferably of the opposing valence), the procedure was repeated.

Directly after recording, protagonists rated the intensity of the emotions they had felt during retelling their event on a list of the following 21 emotional adjectives: angry, mad, furious, sad, downhearted, grieved, afraid, alarmed, worried, disgusted, nauseated, sickened, happy, glad, delighted, proud, productive, satisfied, relaxed, calm, and easygoing; rated on a scale ranged from 1 (*not at all*) to 5 (*extremely*). Emotional adjectives can be categorized according to valence (negative vs. positive affect) or discrete emotion (anger, sadness, fear, disgust, happiness, pride, relaxation).

In this initial stage, we were able to produce a total of 35 neutral and 57 emotional films from 35 protagonists.^3^ Eleven recordings could not be used because the protagonists did not show sufficient emotional involvement and expressivity (nine protagonists) or due to technical problems during the videotaping (two protagonists).

##### Pre-selection and editing of the films

After each session, videotapes were pre-selected by the first author on the basis of the following prerequisites: (1) the protagonist reported a mix of one primary emotion (at a moderate level of intensity; ≥ 3) and at least one secondary emotion of the same valence (at a lower level of intensity), and (2) the protagonist displayed apparent facial and vocal expressions that were in accordance with the protagonists’ self-reported emotions and the film topic. Films that matched these criteria were edited to a length between 1:30 and 2:00 min.

##### Rating of edited film clips

A few days later, protagonists returned to the laboratory to view the edited version of their clip(s) and rated the emotions they had felt during the parts of the narration displayed in the edited film clip on the list of 21 emotional adjectives described above. In addition, protagonists were asked to rate their general emotional involvement on a five-point Likert scale ranging from 1 (*not at all involved*) to 5 (*extremely involved*). Importantly, these ratings highly corresponded with the self-ratings for the original version of the video (*M*_*ICC*_ = .86). The protagonist self-ratings pertaining to the edited film clips were thus used as criteria for computing emotion perception and emotional congruence scores (see below).

Finally, protagonists completed five questions concerning the validity of the recording situation (e.g., “How would you describe this event in your daily life, for example to a familiar person like a friend or a colleague?”; 1 = *completely different* to 7 = *exactly the same*), and three questions concerning the representativeness of the edited film clip in relation to the original recording (e.g., “How well could another person understand the content of the whole situation if he or she had just seen the edited clip?”; 1 = *not at all* to 7 = *very well*; see Supplementary Material, Table SM1 for descriptive statistics).

##### Film selection

Based on our past work, we subsequently utilized a three-step procedure to select appropriate film clips for the validation study (e.g., Kunzmann et al., 2018; Wieck & Kunzmann, 2015).

##### Emotional expressivity

To ensure that the protagonists’ narrations and self-reported emotions matched their nonverbal emotional expressions, two independent trained raters coded the silent film clips for the intensity of emotional expressions pertaining to six basic emotions (anger, sadness, fear, disgust, contempt, happiness; response scale ranged from 1 = *not at all* to 5 = *extremely*) and the intensity of protagonists’ general emotional expressivity, using a coding system from Ekman and Friesen (2003) that we had adapted in our earlier work (Wieck & Kunzmann, 2015). Prior to each film clip, the raters were presented with a neutral picture of the respective protagonist to take idiosyncratic facial characteristics into account. After an intensive training, about 20% of the stimuli were double coded. Agreements between each rater and protagonists’ self-reports concerning the six emotions (Rater 1, ICC = .77; Rater 2, ICC = .76); as well as interrater reliabilities between the two raters (ICC_R1R2_ = .86) were satisfactory.

Film clips were excluded if (a) the protagonist did not express any emotion in the face and, thus, the film was classified as expressively neutral by the raters, and (b) the raters did not agree on the valence ratings (e.g., Rater 1 coded the protagonists’ expression as positive, while Rater 2 coded it as negative). In total, 23 of the 57 film clips had to be excluded, leaving 34 clips for further processing.

##### Protagonists’ characteristics

Past research has suggested that film protagonists’ personality characteristics and outer appearance can influence a perceiver’s empathic responses (Müller et al., 2013) and most likely related competencies such as emotion perception and sympathy. Further, it has been shown that persons’ likability can bias others’ behavior insofar that likeable people arouse more appetence and less avoidance than less likeable people (Herkner, 2004). To determine if our protagonists were rated as typical on these characteristics, we conducted an online survey. An independent sample of 73 adults ranging in age from 20 to 79 years were presented with 35 pictures of the protagonists’ neutral expressions and evaluated each protagonist according to perceived age, attractiveness, intelligence, likability, Big Five personality traits, agency and communion. Participants were instructed to take the protagonists’ age into account when judging the above listed characteristics (response scale ranged from – 2 = *below average* to 2 = *above average*).

Film clips were retained if the sample’s average ratings deviated from 0 ("average") by less than ±1 scale point for a characteristic. In total, we excluded three film clips from three protagonists (see Supplementary Material, Table SM2 for sample characteristics and Table SM3 for descriptive statistics).

##### Content of the reported situation

In a last step, two independent raters evaluated the content of protagonists’ reported situations according to the following features: (1) topics’ age-relevance, (2) topics’ professional relevance, (3) narrations’ comprehensibility, (4) disruptive protagonists’ dialect, and (5) disruptive film cuts. Once again, 20% of the stimuli were double coded. Interrater reliabilities were satisfactory for each of the features (age- and professional relevance, κ = .68; comprehensibility/dialect/cuts, ICC = .77)

First, given that emotion perception is enhanced when the situation reported is particular meaningful for one’s own age group (e.g., Wieck & Kunzmann, 2015; Zhang et al., 2013), we tried to avoid any strongly age-specific film topics that could lead to age biases. Thus, the raters coded whether the topic reported is of particular relevance for a particular age group and, if so, which age group (young, middle-aged, or older workers) this applies to. Two film clips were flagged and excluded.

Second, in order to make the film clips applicable for a wide range of occupations, the raters coded whether the topic is typical for a particular occupational group (e.g., situation would only be encountered in schools) vs. applicable for many occupations (e.g., conflict with supervisor). Since the selected films are considered balanced with regard to their professional relevance, no film clip needed to be excluded.

Lastly, the raters judged further formal characteristics, namely the comprehensibility of the narrative, potential detrimental effects of the protagonists’ dialect on participants' judgment and disruptive cuts during the film. Film clips were excluded if comprehensibility was rated ≤ 3 (response scale ranged from 1 = *not at all comprehensible* to 5 = *extremely comprehensible*), participants' dialect ratings deviated from 0 ("neutral") by more than ±1 scale point, and film cuts were rated ≥ 2 (response scale ranged from 1 = *not at all disruptive* to 5 = *extremely disruptive*). Based on these ratings, three further film clips were excluded. Descriptive statistics are described in Table SM4 in the Supplementary Material.

##### The final film set

At the end of this three-step process, we were able to select a total of 26 film clips of 20 different protagonists varying in emotional valence (11 positive and 15 negative films) and the work situation reported (i.e., situations typical for a certain occupational group vs. applicable equally for many occupations). The final sample of protagonists ranged in age from 20 to 64 years (M = 40.95, *SD* = 14.04, 60% female). Of the sample, eight protagonists were relatively young (20–31 years, *M* = 27.38, *SD* = 3.62; 62.5% women), six middle-aged (36–50 years, *M* = 41.17, *SD* = 5.15; 66.7% women), and six relatively old (56–64 years, *M* = 59, *SD* = 3.95; 50% women). Protagonists were predominantly highly educated with 61.9% having a university degree and worked in various occupational functions and sectors, including healthcare, education, office and administration, finances, and arts and media. Mean tenure in the current occupation was 16.80 years (*SD* = 15.19) and mean tenure in the current organization was 8.45 years (*SD* = 9.42).

#### Participants

The sample comprised 128 employees ranging in age from 19 to 65 years (M = 42.60, *SD* = 12.46; 53.1% female). Participants were recruited in a midsize German city via newspapers and online ads and through the department’s participant pool. The sample was stratified by age and gender with an equal number of participants in three age groups (young, middle-aged, and older workers). Participants were highly educated with 63.3% having a university degree and worked in various economic sectors (e.g., healthcare, education, engineering). Their average occupational tenure was 19.39 years (*SD* = 13.16) and their average organizational tenure was 8.13 years (*SD* = 9.35). Of the sample, 36.7% worked on average more than 8 h per day, 42.2% worked 6–8 h per day, and 21.1% worked less than 6 h per day. Participants’ characteristics (i.e., age, gender, education, profession, and occupational and organizational tenure) roughly correspond with those of the film protagonists. As incentive for participation, participants received 6 euros/hour. All participants gave written informed consent, and experimental methods were approved by the ethics committee of the medical faculty of the University of Leipzig.

#### Procedure and design

The study involved an online survey of about one hour and a subsequent laboratory session of two hours. The online survey included demographic questions along with some further measures not included in this report. In the laboratory session, groups of two to nine participants were seated in cubicles and asked to wear headphones. To limit participant burden, we split the 26 film clips into two equal sets of 13 clips that were balanced in terms of protagonist age, gender, and film valence; participants were randomly assigned to view one of the sets. All participants were instructed to watch one neutral film of a man thinking aloud about his way from home to the laboratory, and, subsequently, 13 emotional films that were presented in two blocks, each presented in randomized order, on a 21-in. computer screen.

After each film clip, participants rated the intensity of the protagonist’s emotions (used to calculate emotion perception) and the intensity of their own emotions that they had felt during the film (as a measure of emotional potency, emotional congruence and sympathy) using the same adjective list as our protagonists used (see above). The self-report adjective list for the participants included three additional adjectives to assess sympathy, that is, sympathetic, moved, and compassionate. At the end of the session, participants were debriefed and received monetary compensation for both sessions.

#### Measures

Descriptive statistics for all empathy facets are depicted in Table 1.

**Table 1:** Characteristics and film-specific scores for emotional reactivity, emotional congruence, sympathy, and emotion perception of 26 Film stimuli used in Study 1

Film characteristics					Emotional Reactivity^a^	Emotional Congruence^b^	Sympathy^c^	Emotion Perception^b^
ID	Age	Gender	Valence (primary emotion)	Film topic	*M (SD)*	*M (SD)*	*M (SD)*	*M (SD)*
40	26	M	P (Ha)	Positive feedback from a business costumer	2.74 (1.1)	.57 (.30)	2.73 (1.02)	.88 (.05)
**43**	**28**	**M**	**P (Ha)**	**Kindergarten child gave a sign of warm affection**	**3.16 (1.1)**	**.66 (.21)**	**3.29 (.93)**	**.87 (.09)**
14	31	F	P (Pr)	Winning a best film award after an exhausting working period	2.52 (1.0)	.57 (.24)	3.20 (.93)	.88 (.08)
41	25	F	P (Ha)	Positive supervisor feedback after a short period of work	2.53 (1.0)	.52 (.27)	2.68 (.87)	.80 (.14)
39	30	M	N (Dg)	Unpleasant odor during a surgery	2.30 (1.1)	.33 (.32)	2.90 (.98)	.56 (.25)
46	20	F	N (Ag)	Derogatory treatment by a colleague	1.98 (.87)	.12 (.32)	2.66 (.89)	.53 (.24)
16	29	F	N (Sa)	Cold-hearted behavior of colleagues	2.46 (.95)	.37 (.28)	3.44 (.97)	.78 (.10)
**24**	**30**	**F**	**N (Ag)**	**Insinuating behavior of the trainer**	**2.83 (1.2)**	**.35 (.41)**	**3.00 (1.0)**	**.71 (.10)**
41	25	F	N (Ag)	Officers’ unfair behavior towards a client	2.32 (1.1)	.26 (.32)	2.36 (.98)	.60 (.18)
**17**	**42**	**F**	**P (Pr)**	**Autistic child spoke for the first time**	**2.50 (.99)**	**.55 (.26)**	**3.44 (1.0)**	**.82 (.07)**
42	41	F	P (Ha)	Valued colleague got an extension of his contract after long struggle	3.43 (.97)	.49 (.22)	3.57 (.89)	.57 (.25)
07	42	M	P (Ha)	Winning a legal dispute against the employer	2.27 (1.0)	.23 (.28)	2.39 (1.08)	.66 (.25)
**15**	**36**	**M**	**N (Ax)**	**Rebellion of prisoners**	**2.77 (1.1)**	**.40 (.38)**	**2.87 (1.05)**	**.74 (.13)**
12	36	F	N (Ag)	Stressful work situation and lack of appreciation	2.13 (.96)	.49 (.32)	2.78 (1.04)	.62 (.13)
42	41	F	N (Sa)	Death of a child after attempt at resuscitation	3.81 (.92)	.61 (.17)	4.49 (.74)	.63 (.13)
27	50	F	N (Ag)	Unfair behavior of a colleague	2.41 (1.1)	.30 (.28)	2.69 (.97)	.58 (.15)
07	42	M	N (Ag)	Unjustified insinuation by the supervisor	2.75 (1.0)	.36 (.31)	3.11 (.97)	.65 (.18)
**31**	**56**	**M**	**P (Ha)**	**Hilarious atmosphere at the clients’ business opening**	**3.35 (1.2)**	**.68 (.25)**	**2.65 (.90)**	**.90 (.10)**
35	58	F	P (Sa)	Appreciative behavior of the parents of two children	2.49 (.94)	.33 (.31)	3.14 (1.02)	.66 (.16)
30	64	F	P (Pr)	Hilarious atmosphere with a critically ill patient	2.24 (1.0)	.54 (.27)	3.31 (.94)	.79 (.11)
26	64	M	P (Pr)	Interested pupils during an exhibition tour	2.17 (1.0)	.55 (.28)	2.90 (1.07)	.84 (.08)
**35**	**58**	**F**	**N (Sa)**	**Bullied pupil commits an act of desperation**	**3.21 (1.1)**	**.44 (.20)**	**4.03 (1.0)**	**.47 (.09)**
30	64	F	N (Sa)	Death of a critically ill patient	2.61 (.93)	.46 (.28)	3.63 (.82)	.66 (.19)
23	56	M	N (Ag)	Unintentional solo performance during a concert	1.88 (1.1)	.10 (.39)	2.68 (1.0)	.74 (.14)
23	56	M	N (Ag)	Forgotten sheet of notes during a concert performance	2.41 (.76)	– .17 (.33)	2.47 (1.04)	.63 (.20)

*Note. M* male protagonist, *F* female protagonist, *P* positive film valence, *N* negative film valence, *Ha* Happiness, *Pr* Pride, *Dg* Disgust, *Ag* Anger, *Sa* Sadness, *Ax* Anxiety

^a^ Response scale ranged from 1 (*not at all*) to 5 (*extremely*). Reactivity indices were calculated by aggregating the three corresponding items of the respective film clips' primary emotion. ^b^ Scores refer to raw intraclass-correlation-coefficients. ^c^ Response scale ranged from 1 (*not at all*) to 5 (*extremely*). Film set deployed in Study 2 (*N* = 6) is marked bold.

##### Emotional reactivity

In order to test if the newly developed film clips are potent to elicit emotional reactions in the participants, a reactivity score was calculated for each film clip by aggregating the three corresponding items of the film’s primary emotion (e.g., participants’ sadness reactivity in response to a primarily sad film).^4^ We considered the theoretical mean of the response scale (i.e., 2.5) as a moderate level of emotional reaction.

##### Emotional congruence

As a measure of emotional congruence, we computed the two-way random, consistency, single-rating measures intraclass correlation coefficients (ICCs) between the participants’ self-ratings of their emotions during the film clip and the protagonists’ self-ratings of their own emotions for the 21 emotional adjectives.^5^ For the statistical analyses, all ICCs were Fisher *r-*to*-Z* transformed to be normally distributed for subsequent analyses (Fisher, 1954). To facilitate interpretation, however, the findings are always presented as the original correlation coefficients.

##### Sympathy

A sympathy score was calculated for each film clip by aggregating the three items, sympathetic, moved, and compassionate, from the self-reported emotion checklist. The theoretical mean of the response scale (i.e., 2.5) is considered as a moderate degree of experienced sympathy.

##### Emotion perception

As a performance-based measure of emotion perception, we computed the two-way random, consistency, single-rating measures ICCs between the protagonists’ self-reported emotions and the participants’ other-reported emotions for each film clip. Again, the ICCs were *r-*to-*Z* transformed for the statistical analyses, but are below reported in terms of original ICCs for better interpretability.

Cutoffs for ratings of convergence-based method (i.e., emotional congruence and emotion perception) are considered poor for values less than .20, fair for values between .21 and .40, moderate for values between .41 and .60, substantial for values .61 and .80, and excellent for values between .81 and 1.0 (Landis & Koch, 1977).

### Statistical analyses

Results are reported in two sections. In the first section, we computed the psychometric characteristics of our newly developed film clips. Specifically, item (i.e., film clip) analyses were conducted per empathy facet that included descriptive statistics (i.e., mean and standard deviation) to examine the level of difficulty/ intensity (for emotional congruence and emotion perception), internal consistency estimates, as well as the potency to elicit emotional reactions (for emotional reactivity and for sympathy). As for internal consistency, in addition to the established reliability index, Cronbach's alpha, the lower bound of Guttman's lambda-2 (Guttman, 1945) is reported.^6^ In the second section, we tested the effects of protagonists’ and participants’ characteristics as well as film clips’ valence on the three different facets of empathy. To this end, we calculated three separate multilevel models for each of the three outcomes: emotional congruence, sympathy, and emotion perception. To implement the models, we used the *lme4* package (Bates et al., 2015) in R (R Development Core Team, 2019). Film-level scores across all 26 film clips of the three facets of empathy served as level-1 variables that were nested in participants (level 2). To account for potential curvilinear age effects, we included both linear and quadratic protagonist age terms. At the within-subjects level (level 1), we included protagonists’ age, protagonists’ age-squared, protagonists’ gender (0 = male, 1 = female), and film clips’ valence (0 = negative, 1 = positive) as predictors. Participants’ age, participants’ age-squared, and participants’ gender were specified as predictors at the between-subjects level (level 2). Except for the dummy variables, all predictors were grand-mean centered (Enders & Tofighi, 2007) before entering them into the model. As participants were assigned to only one film set, all models accounted for film set. We further specified two cross-level interactions, namely protagonists’ gender x participants’ gender and protagonists’ age x participants’ age.

### Results

#### Preliminary results

As depicted in Table 4, intercorrelations among the three facets of empathy show that emotional congruence is positively related to sympathy and emotion perception, while emotion perception is unrelated to sympathy.

#### Psychometric characteristics

Table 1 depicted the film-specific means and standard deviations for emotional reactivity and the three facets of empathy–emotional congruence, sympathy, and emotion perception.

##### Emotional reactivity

As expected, the mean score across all the 26 film clips was moderate, *M* = 2.61 (*SD* = .63). Although ten out of 26 films elicited only small emotional reactions (i.e., score < 2.5), the remaining 16 film clips elicited moderate-to-high emotional reactions in the test taker. Accordingly, most of the film clips fulfill an important requirement for measuring empathy. Internal consistency across the 26 film clips was good, α = .86 (λ_2_ = .87).

##### Emotional congruence

The average score of emotional congruence across the 26 films was fair, *M* = .39 (*SD* = .15). Although 11 out of 26 film clips ranged from poor to low ICCs, indicating that participants shared some of the protagonists’ emotional profiles less, the remaining 15 film clips had moderate to substantial film-specific ICCs of emotional congruence. The internal consistency of emotional congruence can be considered good (α = .77; λ_2_ = .79).

##### Sympathy

Consistent with prior work**,** the mean score of sympathy was moderate, *M* = 3.04 (*SD* = .64). Internal consistency can be considered as good, α = .89 (λ_2_ = .90), confirming that our film clips that were designed to measure sympathy actually do so.

##### Emotion perception

Across the 26 film clips, the average score of emotion perception was moderate (*M* = .70, *SD* = 07) and comparable with studies using similar film-based tasks (Kunzmann et al., 2018; Wieck & Kunzmann, 2017). All film-specific ICCs for emotion perception were positive and ranged from moderate to high (Landis & Koch, 1977), indicating that participants were generally able to identify protagonists’ emotional profiles. Reliability across the 26 film clips was satisfactory (α = .70; λ_2_ = .71), suggesting that emotion perception can be analyzed on the aggregate level.

### Effects of protagonists’, participants’ and film clips’ characteristics on the three facets of empathy

Table 2 summarizes the results of the three multilevel models.

**Table 2:** Multilevel models for emotional congruence, sympathy, and emotion perception across 26 film clips in Study 1

	Emotional congruence			Sympathy			Emotion perception		
	Estimate	*SE*	95% CI	Estimate	*SE*	95% CI	Estimate	*SE*	95% CI
**Intercept**	**.352**	**.045**	**.264; .440**	**2.929**	**.123**	**2.686; 3.173**	**.919**	**.031**	**.859; .979**
**Level 1**									
Protagonists’ age	– .010	.008	– .026; .004	**.084**	**.016**	**.052, .115**	**– .030**	**.006**	**– .042, – .018**
Protagonists’ age^2^	.008	.007	– .006; .022	**– .054**	**.015**	**– .084, – .025**	**.026**	**.006**	**.015, .037**
Protagonists’ gender	– .027	.031	– .088; .034	**.286**	**.066**	**.157, .415**	**– .213**	**.025**	**– .262, – .163**
Valence	**.323**	**.021**	**.282; .364**	.017	.044	– .070, .104	**.387**	**.017**	**.354, .420**
**Level 2**									
Participants’ age	– .021	.015	– .050; .009	**.094**	**.045**	**.005; .183**	**– .024**	**.009**	**– .042; – .005**
Participants’ age^2^	.024	.013	– .002; .050	.037	.039	– .040; .114	– .009	.008	– .025; .007
Participants’ gender	– .008	.045	– .097; .081	– .085	.123	– .328; .158	.023	.032	– .040; .085
**Cross-level interactions**									
Protagonists’ Age x Participants’ Age	.002	.006	– .010; .013	– .006	.013	– .031; .019	.008	.005	– .001; .018
Protagonists’ Gender x Participants’ Gender	.022	.043	– .062; .105	.140	.090	– .037; .317	.005	.035	– .062; .073
**Residual variances**	**.174**	**.041**	**.402; .432**	**.778**	**.082**	**.852; .914**	**.114**	**.035**	**.327; .351**

*Note*. *N* = 26 film clips rated by 128 participants. Protagonists’ age and participants’ age are scaled in decades (i.e., one unit represents 10 years).

Gender was coded as 0 = male and 1 = female. Valence was coded as 0 = negative and 1 = positive. Estimate scores of emotional congruence and emotion perception refer to Fisher-z transformed ICCs. Significant coefficients at *p* < .05 are bolded.

#### Emotional congruence

The analyses revealed that the film clips’ valence was the only significant predictor, indicating that emotional congruence was higher during positive than negative films. Neither level-2 predictors nor cross-level interactions were significant.

#### Sympathy

Analyses indicated that protagonists’ age, age-squared, and gender as well as participants’ age were significant predictors. More specifically, protagonists’ age had a positive linear effect, while protagonists' age-squared had a negative effect, suggesting that sympathy towards middle-aged protagonists was higher than sympathy towards both younger and older protagonists. Further, participants reported greater sympathy for older than younger protagonists. Analyses also indicated that female protagonists elicited greater sympathy than male protagonists, and, as predicted, older participants reported higher sympathy towards the film protagonists than younger adults. No cross-level interaction became significant.

#### Emotion perception

All level-1 predictors for main effects were significant. Specifically, analyses revealed that protagonists’ age had a negative linear effect, while protagonists' age-squared had a positive effect, suggesting younger protagonists’ emotions were more easily perceived than those of middle-aged or older protagonists in our sample. Analyses further revealed that men’s emotions were more accurately perceived than women’s emotions, and emotion perception was better if the protagonists talked about positive, as compared with negative, events. Furthermore, the level-2 predictor participants’ age was significant. As predicted, and consistent with previous research, older adults performed worse at perceiving others’ emotions than young adults. No cross-level interaction became significant.

## Discussion

By employing the relived emotion paradigm, we were able to develop a new set of 26 ecologically valid film clips, allowing us to assess three facets of empathy, that is, emotional congruence, sympathy, and emotion perception, in vivo. The findings suggest that the majority of film clips successfully elicited emotional reactions of moderate intensity in the test takers, offering the possibility to assess emotional congruence and sympathy in more ecologically valid ways than most prior measures do. With a Cronbach’s alpha between .70 and .89, internal consistencies in the three empathy facets were acceptable, consistent with those in past studies using the same paradigm (e.g., Wieck & Kunzmann, 2015). In line with prior work (e.g., Katzorreck & Kunzmann, 2018), the new film clips showed a moderate average level of difficulty for emotion perception and a moderate level of experienced sympathy, while mean scores for emotional congruence were relatively low. While the focus in emotion perception lies particularly on another person's emotions and can proceed almost independently of the test taker's own emotional state, the process of emotional congruence requires more coordination and fine-tuning between one's own and another person's emotions. Similarly, sharing another person's emotions is more demanding as it requires an additional skill, emotion regulation, such as to be able to maintain emotional reactions in response to other persons’ distress within a tolerable range (e.g., Eisenberg et al., 1994). Thus, it is plausibly that emotional congruence typically is relatively lower than emotion perception.

As predicted, our film-based analyses suggest that protagonists' age, gender, and film clips’ valence had effects on empathy. To begin, consistent with past work (e.g., Ebner et al., 2011; Ruffman et al., 2020), older protagonists’ emotions were most difficult to perceive. In contrast to our predictions, however, participants reported the least sympathy towards younger protagonists. One possible explanation could be that older adults are perceived as more vulnerable than younger adults and are accordingly treated with more concern and warmth (Fingerman & Charles, 2010). As to the effects of participants’ age, consistent with past findings, our evidence fits the notion that emotion perception decreases across age groups, emotional congruence is similar, and sympathy increases (Ruffman et al., 2020; Sze, Gyurak, et al., 2012b; Wieck & Kunzmann, 2015).

As to gender, whereas emotions of male protagonists were easier to perceive than female protagonists’ emotions, sympathy was higher for female, as compared with male, protagonists. At this point, it is difficult to explain these findings, however, in the present study, male and female protagonists did not differ in the intensity of their emotional expressions. Yet, we cannot exclude the possibility that male protagonists’ emotional expressions were less complex or diverse, making them easier to read and dampening sympathy towards them. Notably, we found gender differences only for protagonists and not for test takers. This is consistent with past work with test-based measures of empathy (Fischer et al., 2018).

Finally, as predicted and consistent with prior work, emotional congruence and emotion perception were higher during positive films.

In sum, the present film set allows to assess three facets of empathy within emotionally charged real-life job situations on the basis of film-based stimuli with satisfactory psychometric properties. Interested researchers can choose up to 26 film clips, dependent on the facet of empathy that is of interest. It is also noteworthy that our test material allows the systematic investigation of situation-specific characteristics, particularly the protagonists’ age, gender, or dominant emotional state (positive vs. negative).

Nevertheless, in some research settings, utilization of a large set of films might not be feasible or might be considered too onerous. Examples are online studies, studies with a large battery of instruments, and studies with special populations, such as older employees. In these settings, the availability of a short, validated version of the empathy task might be desirable. To provide organizational researchers with a reliable and valid toolkit suitable for capturing all three facets of empathy with the same film set, we selected a brief “best-of” of the film clips generated in Study 1 and examined their psychometric characteristics and construct validity in a second study.

## Study 2

In Study 2, we selected a fixed set of six film clips from the larger pool of films developed and validated in Study 1. This film set was presented to an independent sample of employees in an online study with a follow-up session after 4 weeks. A first goal was to test for two forms of reliability, namely, internal consistency and test–retest reliability. A second goal was to obtain initial information about construct validity of the short film set. To this end, we addressed the association between the three empathy dimensions and alternative trait self-reports and test-based measures of empathy, as well as their differential associations with cognitive functioning. Finally, a third goal was to replicate the findings regarding the effects of the age and gender on empathy dimensions reported in Study 1.

### Reliability

Reviewing contemporary research on empathy measures indicated that, compared to internal consistency, retest reliability plays a considerably minor role. Aside from the inconvenience of testing the same sample twice, retest reliability might have been neglected because many researchers apparently assume that different measures of reliability are interchangeable (McCrae et al., 2011). Yet, internal consistency, which refers to the coherence (or redundancy) of the components of an measurement instrument, is conceptually independent of retest reliability, which reflects the extent to which similar scores are obtained when the instrument is administered on different occasions separated by an interval (McCrae et al., 2011). The few studies that have examined test–retest reliabilities for test-based measures of emotion perception, report reliabilities ranging from *r*_*tt*_ = .61 (e.g., MSCEIT subscale emotion perception, Raymond et al., 2014) to *r*_*tt*_ = .84 (e.g., DANVA, Nowicki & Duke, 1994). Usually, the time intervals between the two measurement points are relatively short, ranging from 2 to 9 weeks. Given that the present retest interval was also relatively short, lasting 4 weeks, and our conceptualization of empathy as a relatively stable person-related ability and trait (e.g. Mast & Ickes, 2007), we predicted that both internal consistency and test-retest reliability should be moderate to high.

#### Construct validity

#### Relation to existing measures

In the past, researchers noted that correlations among different tests – especially those for emotion perception – can be low (Boone & Schlegel, 2016). One explanation for this finding might be that, as mentioned above, most traditional tests of emotion perception include unimodal and static stimuli, and therefore measure rather narrow and specific skills. Given that our film-based tasks intended to be a measure of broad empathic competencies that subsumes narrower and more modality-specific skills, we expected to find positive correlations between our own and other researchers' measures of the same or closely related empathy facet measured with a similar test. We thus predicted that emotion perception would be most highly correlated with test-based measures assessing the same competency. As emotional congruence and sympathy were measured via self-evaluation, we expected low to moderate correlations with trait self-report questionnaires.

#### Relation to cognitive functioning

Past work suggests that the cognitive and affective dimensions of empathy rely on different mechanisms. For instance, emotion perception has been found to require both basic fluid cognitive abilities (e.g., processing speed; Schlegel et al., 2020; Wieck & Kunzmann, 2015) as well as pragmatic cognitive abilities (e.g., verbal ability, Kunzmann et al., 2018). Higher processing speed might especially increase performance when perceiving dynamic and complex emotional expressions in which many and brief (non)verbal cues must be processed at the same time. Better verbal knowledge might benefit emotion perception performance because it is likely related to a more nuanced knowledge about the different emotion words and their meaning. By contrast, emotional congruence and sympathy have been thought to rely less on cognitive functioning and more on emotional and motivational processes, including the motivation to care for others or the ability to regulate self-focused negative emotions (Losoya & Eisenberg, 2001; Thompson et al., 2019). Thus, we predicted that emotion perception is positively associated with different facets of basic cognitive abilities, while emotional congruence and sympathy should be unrelated.

### Age and gender effects

Finally, since multidirectional age effects in empathy but no effects of gender were found for the entire set of film clips in Study 1, we expected to replicate this pattern in Study 2.

### Method

#### Participants

Study participants were recruited through the German survey division of Respondi (© respondi AG, Köln, Germany) from a nonprobability panel of people that regularly participate in online surveys. The study was announced as investigating emotional experiences and emotional competence, consisting of two survey sessions to be completed with an interval of 4 weeks. Participants received monetary compensation for their attendance. Of the 130 employed adults aged 20 to 64 years (*M* = 40.93, *SD* = 11.66, 46.2% female) that completed the first survey (Time 1), a total of 99 employees ranging in age from 21 to 64 years (M = 43, *SD* = 11.41) participated and completed the second survey after 4 weeks (Time 2). Accordingly, the dropout of study participants between the two time points was 23%. Dropouts did not differ in age and gender from the final sample (*p* > .10). The final sample was stratified by age (19.2% between 21 and 30 years, 22.2% between 31 and 40 years, 28.3% between 41 and 50 years, and 25.3% between 51 and 60 years, and 5.1% 61 years and older) and gender (44.4% female). Of the sample, 38.4% held a university degree, 59.6% had vocational training, and 2% had no further training after secondary school. Participants worked in various occupational functions and sectors (e.g., health and social welfare, education, ICT, office and administration). Mean tenure in the current occupation was 20.91 years (*SD* = 11.88) and mean tenure in the current organization was 10.43 years (*SD* = 9.28). Similar to Study 1, participants’ characteristics roughly correspond with those of the film protagonists.

#### Procedure and Design

Both surveys were administered in German using the Unipark online survey tool and each took about 30 min to complete. Participants were instructed to complete each survey in a quiet environment.

The survey at Time 1 contained a demographic questionnaire and a selected set of our newly developed film clips measuring emotional congruence, sympathy, and emotion perception (see below). All participants then completed a measure of crystallized cognition and a self-report scale assessing different facets of empathy. In the follow-up assessment 4 weeks later, participants completed the same film clips to assess the three empathy facets as at Time 1. In addition, participants were asked to complete a measure of fluid cognition as well as a performance-based test assessing emotion perception.

#### Short Film Set to Assess Three Facets of Empathy

In order to assess the three empathy components time-efficiently and with the same set of stimuli, we carefully selected six of our newly developed film clips that (1) elicited emotional reactions of moderate intensity in the test takers, (2) showed at least a moderate average level of difficulty to capture individual differences in a reliable way, and (3) were balanced in terms of protagonist age, gender, and film valence (see Table 1 for the selected film clips; displayed in bold). We chose six films as this makes it possible to administer them in a reasonable amount of time (20 min) while ensuring some variety in the relevant film characteristics.

As in Study 1, participants were instructed to watch the six film clips that were presented in randomized order. After each film clip, they rated the intensity of the protagonist’s emotions (used to calculate emotion perception) and the intensity of their own emotions that they had felt during the film (used to calculate emotional congruence and sympathy) using the same adjective list as our protagonists used (see above). The same procedure as in Study 1 was used to calculate emotional congruence, sympathy, and emotion perception.

### Measures to test construct validity

Descriptive statistics for all the variables measured in Study 2 are displayed in Tables 3 and 4.

**Table 3:** Internal consistency, descriptives at Time 1 and Time 2, and test–retest reliability for three empathy facets in Study 2

Measure	α (λ2)		*M (SD)*		Test-Retest
	T1	T2	T1	T2	*r*_*tt*_
Emotional congruence ^a^	.80 (.81)	.80 (.81)	.49 (.24)	.46 (.24)	.68^**^
Sympathy ^b^	.81 (.82)	.89 (.90)	3.05 (.75)	2.94 (.86)	.70^**^
Emotion perception ^a^	.88 (.89)	.90 (.90)	.62 (.27)	.61 (.28)	.81^**^

*Note. T1* Time 1, *T2* Time 2 (4-week interval); Internal consistency denoted as Cronbach’s Alpha, Guttman’s Lambda-2 reported in brackets

^a^ Scores refer to raw intraclass-correlation-coefficients.

^b^ Response scale ranged from 1 (*not at all*) to 5 (*extremely*)

**Table 4:** Descriptive statistics and correlations between variables in Study 1 (above the diagonal) and Study 2 (below the diagonal)

Measure	M (SD)	1	2	3	4	5	6	7	8	9	10	11	12
1. Emotional congruence	-	-	.45^**^	.34^**^	– .05	– .03	-	-	-	-	-	-	-
2. Sympathy	-	.56^**^	-	– .15	.21^*^	– .01	-	-	-	-	-	-	-
3. Emotion perception	-	.63^**^	.14	-	– .24^**^	.11	-	-	-	-	-	-	-
4. Participant age	43 (11.4)	.25^*^	.31^**^	– .23^*^	-	– .06	-	-	-	-	-	-	-
5. Participant gender	-	.22^*^	– .08	.12	.08	-	-	-	-	-	-	-	-
6. FACES ^a^	72.4 (12.52)	.19	– .08	.39^**^	– .07	.22^**^	-	-	-	-	-	-	-
7. ECQ cognitive ability	2.98 (0.52)	.29^**^	.14	.40^**^	.23^*^	.16	.12	-	-	-	-	-	-
8. ECQ affective reactivity	3.04 (0.55)	.40^**^	.25^*^	.44^**^	.30^**^	.11	.22^*^	.63^**^	-	-	-	-	-
9. ECQ affective ability	3.00 (0.57)	.35^**^	.25^*^	.40^**^	.25^*^	.20^*^	.18	.69^**^	.68^**^	-	-	-	-
10. ECQ affective drive	3.12 (0.49)	.39^**^	.20^*^	.48^**^	.26^**^	.18	.22^*^	.48^**^	.64^**^	.55^**^	-	-	-
11. Verbal ability^a^	73.91 (14.13)	.27^**^	.00	.46^**^	.32^**^	.12	.19	.19	.20^*^	.19	.21^*^	-	-
12. Spatial processing speed^a^	34.59 (13.81)	– .18	– .26^*^	.21^*^	– .33^**^	– .16	.17	– .12	– .05	– .14	– .12	.07	-

*Note.* Correlations above the diagonal refer to Study 1 (*N* = 128 using 26 film clips); correlations below the diagonal pertain to Study 2 (*N* = 99 using six film clips). To calculate correlations with the film-based constructs of Study 2, values of the second measurement time point (Time 2) were used. Gender was coded as 0 = male and 1 = female. FACES = Traditional performance-based emotion perception task; ECQ = Empathy Components Questionnaire

^a^ In percent. ^*^p < .05. ^**^ *p* < .01.

#### Self-report of empathy

Participants completed a total of 20 items of the Empathy Components Questionnaire (ECQ, Batchelder et al., 2017) on a scale from 1 (*strongly disagree*) to 4 (*strongly agree*). We used four subscales comprising both cognitive and affective facets of empathy, namely, cognitive ability (i.e., skill, capacity, or potential in perspective-taking and to adopt another’s point of view), affective reactivity (i.e., the tendency to share other’s emotions and feelings), affective ability (i.e., the skill, capacity, or potential in recognizing, being sensitive to, and sharing others’ emotions), and affective drive (i.e., the motivated interest or tendency in recognizing, being sensitive to, and sharing others’ emotions). Internal consistencies for the subscales were largely satisfactory (cognitive ability, α = .63, λ_2_ = .65; affective reactivity, α = .72, λ_2_ = .73; affective ability, α = .73, λ_2_ = .74; affective drive, α = .62, λ_2_ = .64).

#### Traditional test-based emotion perception test

The traditional facial emotion recognition task included 36 colored pictures from the FACES database (Ebner et al., 2010) showing younger, middle-aged, and older adults (50% women) displaying angry, happy, sad, disgusted, neutral, and fearful facial expressions (six pictures per emotion). After presenting each picture for 5s, participants were asked to select one of eight emotion labels (i.e., six basic emotion labels and two distractors, surprise and contempt) to indicate the emotion expressed in the face. Proportion of correct responses was computed, ranging between 17% and 100% in the present sample. Internal consistency can be considered good, α = .74 (λ_2_ = .76).

#### Cognitive functioning

This study included one measure of fluid cognition and one measure of crystallized cognition. As a fluid indicator we chose a spatial processing speed task from the cognitive test battery (*Leistungsprüfsystem,* LP, Horn, 1983). Participants were presented with a set of objects and asked to count the number of surfaces of as many objects as possible in two minutes using a multiple-choice response format. Processing speed is represented by the number of correct answers ranging in the present sample from 7 to 70%. Reliability was good, α = .86 (λ_2_ = .87). As an indicator of crystallized cognition, participants completed a vocabulary test (*Wortschatztest*, Schmidt & Metzler, 1992). In this test, 42 rows, each consisting of six words, are presented. In each row one target word has to be identified among five pseudo-words. Verbal ability, calculated as number of correct responses, ranged from 10 to 93%. Internal consistency across the 42 items was good, α = .91 (λ_2_ = .92).

### Data analysis

To evaluate the psychometric quality of the facet-specific measures of empathy, we analyzed the (1) internal consistency, (2) test–retest reliability, (3) construct validity, and (4) correlations with participants’ age and gender. Similar to Study 1, internal consistency was measured using Cronbach's alpha in addition to Guttman's lambda. The test–retest coefficient (or stability coefficient) was assessed by calculating the Pearson's Product Moment Correlation coefficient. A value of +1 indicates perfect stability, a value of 0 indicates no stability. A good test–retest reliability is defined by correlations of at least *r*_*tt*_ = .70 (Amelang & Schmidt-Atzert, 2006). Finally, construct validity of our empathic constructs was examined by calculating correlations between the three facets of empathy and FACES, ECQ, and cognition. To do so, we first obtained film-specific scores by computing ICCs (for emotional congruence and emotion perception) and average item scores (for sympathy), and then aggregated these scores across all film clips per test taker.

## Results

### Preliminary analyses

As depicted in Table 3, similar to Study 1 and comparable with studies that used the same film-based paradigm to measure empathy (e.g., Wieck & Kunzmann, 2015), mean values of emotion perception were moderate at both time points, while the values of emotional congruence were somewhat lower. Moreover, the film clips elicited a moderate level of experienced sympathy in the test takers. Similar to Study 1, intercorrelations among the three facets of empathy show that emotional congruence is positively related to sympathy and emotion perception, while emotion perception is unrelated to sympathy.

### Internal consistency and test–retest reliability

Reliability analyses performed on emotional congruence, sympathy, and emotion perception indicated good estimates of internal consistency at both measurement time points, suggesting that each of the three components of empathy can be analyzed on the aggregate level (α from .80 to .90; see Table 3). Consistent with our prediction, test–retest correlation coefficients between the three corresponding facets administered at Time 1 and at Time 2 ranged from moderate to high (*r*_*tt*_ from .68 to .81), ensuring that our film-based tasks reliably measure participants' empathy and participants tend to remain stable on their emotional congruence, sympathy, and emotion perception over 4-week interval (see Table SM7 in the Supplementary Material for film-specific test–retest reliability).

### Construct validity

As expected, while emotion perception was positively related to the FACES test, emotional congruence and sympathy were unrelated to this test. Furthermore, with one minor exception, all three facets of empathy were positively associated with the four subscales of the self-reported empathy measure ECQ. As to cognition, higher scores on emotional congruence and emotion perception were related to higher verbal ability, whereas sympathy was unrelated to verbal ability. As expected, higher scores on emotion perception were positively related to processing speed. Surprisingly, sympathy was found to be slightly negatively associated with processing speed. We reasoned that this may be due to age as a confounding factor, given that age is negatively associated with processing speed but positively associated with sympathy. To test this assumption, we calculated partial correlations of sympathy with processing speed while controlling for age. Indeed, the partial correlation between sympathy and processing speed became nonsignificant (*r* = – .11, *p* = .297).

### Correlations of empathy with age and gender

Mostly consistent with previous findings, participants’ age was positively related to emotional congruence and sympathy, but negatively associated with emotion perception (e.g., Kunzmann & Richter, 2009; Sze, Gyurak, et al., 2012b; Wieck & Kunzmann, 2015). In contrast to our predictions, gender was positively related to emotional congruence (0 = male, 1 = female), but unrelated to the other empathic facets, emotion perception and sympathy.

## Discussion

Using a two-wave online study, we demonstrated that our subset of six film-based empathy tasks show acceptable reliability and validity. Internal consistencies of the short film set ranged from .80 to .90, which is comparable with Study 1 and slightly higher than in prior work using the same paradigm to develop film stimuli (Katzorreck & Kunzmann, 2018; Wieck & Kunzmann, 2015). The test–retest reliability of the facet-specific empathy measures ranged from moderate to high (.68 to .81), indicating that the film-based measures of empathy obtained in one session are stable over time. As predicted, and in line with former studies (Ciarrochi et al., 2002; Sze, Goodkind, et al., 2012a) we found that emotion perception correlated positively with FACES, a test-based measure assessing the same competency. Although our film-based measure clearly assesses an individual’s ability to infer others’ emotions, it has its unique features and differs in a number of characteristics from FACES, including the context-richness, multi-modality, as well as the authenticity and complexity of the emotions expressed by the protagonist. It is thus not surprising that the size of the correlation between the two types of emotion perception measures was only moderate. Furthermore, with only one exception, all film-based measures were positively correlated with the subscales of the ECQ, providing further evidence for construct validity of our film measure. Consistent with the idea that emotion perception requires cognitive resources, our results showed that higher perception speed and better verbal abilities substantially contributed to the accurate perception of another person's emotion. As predicted, emotional congruence, a construct with little cognitive load, did not correlate with perceptual speed, but in contrast to our prediction and prior work (Wieck & Kunzmann, 2015) emotional congruence was positively, albeit weakly, related to verbal ability. This link makes sense insofar as test takers needed a nuanced knowledge about the different emotional adjectives and their meaning to indicate their own emotional state while watching the film clips. Somewhat unexpectedly, we found sympathy to be slightly negatively related to perceptual speed. However, controlling this relationship for test takers' age, the link between perceptual speed and sympathy disappears. Largely consistent with our predictions and previous work (e.g., Richter & Kunzmann, 2011; Ruffman et al., 2020; Sze, Gyurak, et al., 2012b), we were able to show that emotion perception was negatively related to age, whereas the affective components of empathy correlated positively with age. In contrast to Study 1, we also found a small gender difference, with women sharing others’ emotions somewhat more than men. Actually, the finding that women are more empathic than men have been mainly observed in self-reports and has been taken as an indication for demand characteristics in a sense that women feel they are expected to be more socially sensitive (Ickes et al., 2000). However, some studies have also reported behavior-based differences, showing for instance that empathy in women is less susceptible to previous unfairness of the observed other (Singer et al., 2006).

## General discussion

During the past decades, empathy has become a widely researched construct in organizational psychology. At the same time, relatively little progress has been made in terms of improving the measurement of empathy, with most studies using either self-reports or test-based measures including static and unimodal stimuli far-fetched from real-life situations and unlikely to evoke emotions in the observer (Clark et al., 2019). In order to complement and expand previous work, we developed and validated a new set of emotion-evoking stimuli that allows measuring empathy in a more ecologically valid way than many existing measures do. By using the relived emotion paradigm, we produced a set of 26 film clips that display people thinking-aloud about autobiographical work situations while experiencing and expressing positive and negative emotions authentically. In Study 1, we provided evidence for the satisfactory psychometric properties of these films, as indicated by a moderate level of difficulty and emotional intensity, as well as high internal consistency. Utilizing a subset of six film clips, in Study 2, we showed that the three facet-specific empathy tests had moderate to high internal consistencies and test–retest reliabilities. We further provided first evidence for the construct validity of the brief empathy measures, showing moderately positive associations with existing self-report and test-based measures of empathy, as well as expected relationships with cognitive functioning. Notably, many of the results were robust and generalized across the present two studies.

Three key findings deserve to be highlighted. First, extending extant measures of empathy, the majority of our film clips are potent to elicit a wide range of self-related emotions (e.g., sadness, anger, happiness) as well as other-related emotions (e.g., sympathy) in the test takers. Notably, in addition to the elicitation of primary emotions ranging from moderate to high intensity, the film clips concurrently evoked secondary emotions of lower intensity and similar valence in the test taker (see Table SM5 and SM6 in the Supplemental Material for detailed information). Crucially, this offers the possibility to cover the breadths of the construct of empathy and thus also to assess the role of affective facets of empathy, largely neglected in previous organizational research, in more ecologically valid ways, that is, in situations in which the test taker is emotionally aroused when confronted with critical events at work.

Second, our findings indicate that scholars should aim for diversity in protagonists and topics when selecting testing materials. Extant measures that include only one age group, gender, or stimulus valence are likely biased since they may over- or underestimate test takers’ empathic responses and competencies. Consequently, in age-comparative work, it is important to balance the protagonists’ age. In gender-comparative work, it is important to use stimulus material depicting men and women. In addition, given that many empathic responses (e.g., emotional congruence) are seemingly more easily evoked if test takers are confronted with positive than negative events, it is important to systematically vary the valence and perhaps even the discrete emotional quality of the testing material.

Third, we identified a “best-of” of six film clips covering a range of different features (i.e., protagonists’ age and gender, film valence) that is suitable to assess cognitive and affective facets simultaneously and in a relatively short time (20 min). These facet-specific measures of empathy are shown to be (a) reliable, as indicated by satisfactory internal consistencies and retest reliabilities, and (b) valid, as indicated by theoretically meaningful correlations with other empathy tests and cognitive functioning. Given the short test-taking time and its satisfactory psychometric properties, this film set can be considered a useful addition to the range of available empathy tests.

## Future applications

An advantage of our newly designed film clips is that many of the situations reported were tailored to work contexts applicable to most jobs. Thus, our film clips are a suitable measure for research studies as well as for applied settings. Such applications might include an analysis of employees’ potential, personnel decisions, and trainings or development interventions. For example, based on empathic responses and competencies, individual profiles of participants’ level can be calculated and used to identify areas of improvement for personalized training.

The results on emotional reactivity further provide the basis to test related socio-emotional competencies, such as individuals' emotion regulation capacity. To accomplish this, either the short film set or a self-generated selection of film clips from the larger set of 26 films with at least a moderate level of emotional reactivity can be used. Future studies can present these films to participants under different emotion regulations instructions. Following past research on emotion regulation (e.g., Scheibe & Blanchard-Fields, 2009; Troy et al., 2013), a subset of the films could be shown with a "natural viewing" instruction (*‘Watch the film carefully’*), while a second, comparable film set could be shown with a "suppression" instruction (*‘Try to hide your feelings so that an outside observer would not know what you are feeling’*) and/or an “amplification” instruction (*‘Try to show your feelings so that an outside observer would know exactly what you are feeling’).* Emotion regulation ability can be operationalized by comparing emotional responses at different levels (subjective experience, physiology, facial expression) across different emotion-regulatory conditions.

## Limitations

Several limitations deserve mentioning. First, future research is needed to provide additional evidence for the psychometric quality of our film set, including their construct and predictive validity. For instance, it could be systematically examined to what extent the film-based measures correlate with tests for other components of social-emotional competence, such as emotion understanding or emotion regulation, as well as with measures of general intelligence and personality. Second, a general limitation of the relived emotions paradigm deployed is that the film protagonists do not report their actual emotional reactions in response to ongoing situation, but reflect on an already experienced event and remember the accompanied emotions in the laboratory. Nevertheless, this paradigm provides a substantial increase in ecological validity of the stimuli compared to existing measures, while still ensuring comparability of the emotional stimuli used to test for empathic responses and competencies across test takers. Finally, given that the film clips were produced in German, their use is largely limited to research in German populations. This limitation is inherent in the development of ecologically valid stimuli, as intelligible spoken language - compared to pseudo-speech sentences used in prior work - represents a key aspect of real-life emotional job situations. At the same, the general film-based paradigm used can be applied to other languages.

## Conclusions

By applying the relived emotion paradigm developed in emotion research, we were able to produce new film clips that allow to assess three dimensions of empathy – that is, emotional congruence, sympathy, and emotion perception, as they occur in real-life work situations. Our results reveal that the film clips evoke a moderate level of emotions in the test taker, enabling their use to assess affective facets of empathy and further important competencies (such as emotion regulation) in vivo in future studies. Organizational research is likely to benefit from this testing material for empathy constructs to advance understanding of how these affect work and organizational outcomes.

## Data Availability

Academic researchers can request using the newly developed film clips and the datasets analyzed during the current study through sending a request to the corresponding author (cornelia.wieck@uni-leipzig.de). A user agreement form signed by a researcher with a valid academic affiliation is required to obtain access to the film clips. The detailed description of film production and selection procedures allows for developing similar films in other languages and cultures.

## Footnotes

1 Buck et al. (2017) introduced the Communication of Affect Receiving Ability Test-Spontaneous, Posed, Regulated (CARAT-SPR) to measure empathy. Although the CARAT-SPR contains film stimuli displaying protagonists' spontaneous facial expressions while responding to emotionally loaded images, this test was only used to assess participants' ability to detect emotions and to differentiate spontaneous, posed, and regulated displays. Accordingly, it is unclear whether the stimuli elicit emotions and thus are suitable to capture the affective facets of empathy.

2 Since the current project was conducted in Western Europe, age, and gender diversity were the most important diversity dimensions to cover (as compared to race and ethnicity which are more salient in other regions).

3 The varying numbers of film clips and protagonists are due to the fact that some protagonists were able to report more than one emotional situation (e.g., one positive and one negative event) from their professional life.

4 Besides measuring emotional reactivity at the level of discrete emotions, it is common to assess individuals' responses in terms of broad dimensions such as valence and arousal. In addition to the film-specific reactivity scores for all primary and secondary discrete emotions, we also provide the aggregated valence-specific intensity ratings per film clip (negative affect and positive affect; see Table SM5 and Table SM6 in the Supplementary Material).

5 Unlike "absolute" ICCs, which take into account the mean-level differences between the protagonists' ratings and the participants' ratings, "consistent" ICCs do not reflect mean-level differences. However, since mean-level differences can be influenced by a person's response behavior (e.g., central tendency bias; Bernieri et al., 1994), we have chosen the consistency measure so that this bias cannot influence our measures.

6 There is an ongoing debate that Cronbach’s alpha as a measure of internal consistency suffers from major problems (Schmitt, 1996; Sijtsma, 2008). Therefore, researchers recommend to report Guttman's Lambda 2 (Guttman, 1945) which is a lower bound to the reliability and equals the true reliability if the components are tau equivalent, as a more accurate estimate of the reliability.
